# Obesogenic Diets Cause Alterations on Proteins and Theirs Post-Translational Modifications in Mouse Brains

**DOI:** 10.1177/11786388211012405

**Published:** 2021-05-03

**Authors:** Valentina Siino, Pia Jensen, Peter James, Sonya Vasto, Antonella Amato, Flavia Mulè, Giulia Accardi, Martin Røssel Larsen

**Affiliations:** 1 Department of Immunotechnology, Lund University, Sweden; 2 Department of Biochemistry and Molecular Biology, PR Group, University of Southern Denmark, Odense, Denmark; 3 Turku Centre for Biotechnology and Åbo Academy University, Turku, Finland; 4 Department of Biological Chemical and Pharmaceutical Sciences and Technologies (STEBICEF), University of Palermo, Palermo, Italy; 5 Institute of Biomedicine and Molecular Immunology ‘Alberto Monroy’ CNR, Palermo, Italy; 6 Department of Biopathology and Medical biotechnologies Pathobiology and Medical Biotechnologies, University of Palermo, Palermo, Italy

**Keywords:** Obesity, nutrition, brain impairment, proteomics, post-translational modifications

## Abstract

Obesity constitutes a major global health threat and is associated with a variety of diseases ranging from metabolic and cardiovascular disease, cancer to neurodegeneration. The hallmarks of neurodegeneration include oxidative stress, proteasome impairment, mitochondrial dysfunction and accumulation of abnormal protein aggregates as well as metabolic alterations. As an example, in post-mortem brain of patients with Alzheimer’s disease (AD), several studies have reported reduction of insulin, insulin-like growth factor 1 and insulin receptor and an increase in tau protein and glycogen-synthase kinase-3β compared to healthy controls suggesting an impairment of metabolism in the AD patient’s brain. Given these lines of evidence, in the present study we investigated brains of mice treated with 2 obesogenic diets, high-fat diet (HFD) and high-glycaemic diet (HGD), compared to mice fed with a standard diet (SD) employing a quantitative mass spectrometry-based approach. Moreover, post-translational modified proteins (phosphorylated and N-linked glycosylated) were studied. The aim of the study was to identify proteins present in the brain that are changing their expression based on the diet given to the mice. We believed that some of these changes would highlight pathways and molecular mechanisms that could link obesity to brain impairment. The results showed in this study suggest that, together with cytoskeletal proteins, mitochondria and metabolic proteins are changing their post-translational status in brains of obese mice. Specifically, proteins involved in metabolic pathways and in mitochondrial functions are mainly downregulated in mice fed with obesogenic diets compared to SD. These changes suggest a reduced metabolism and a lower activity of mitochondria in obese mice. Some of these proteins, such as PGM1 and MCT1 have been shown to be involved in brain impairment as well. These results might shed light on the well-studied correlation between obesity and brain damage. The results presented here are in agreement with previous findings and aim to open new perspectives on the connection between diet-induced obesity and brain impairment.

## Introduction

Obesity is a major health problem worldwide. According to the Eurostat report of 2019, roughly half of the European population (51.6%) had a condition of overweight in 2014. Obesity is mainly caused by environmental factors and life style such as low physical activity and increase in the consumption of high-fat diets. Obesity is a multifactorial disease associated with the aetiology of other disorders such as type 2 diabetes, cardiovascular disease, hepatic steatosis, biliary disease and cancer.^1^ Only in the last 2 decades obesity has been proposed as a risk factor for brain impairment.^2^ Several studies have shown that obese rats exhibit worse performance on learning and memory tasks as well as cognitive dysfunctions compared to non-obese rats.^3,4^ Furthermore, in vivo models have shown that high-fat diet is responsible for neuronal loss and synaptic plasticity impairment.^5,6^ In addition, metabolic syndrome, associated with the development of obesity, type 2 diabetes, hyperinsulinaemia and glucose resistance has been linked to the development of Alzheimer’s disease (AD).^7,8^ An American study conducted on AD postmortem human brains showed a reduction of insulin, insulin-like growth factor 1 and insulin receptor together with an increase in tau protein and glycogen-synthase kinase-3β. The above-mentioned study suggests an impairment of metabolism in the human brain allowing the classification of AD as a neuro-endocrine disorder similar to diabetes mellitus.^7^ Since then, many studies have tried to unveil the possible mechanisms linking the metabolic syndrome to brain dysfunctions. The main hypothesis is that subjects with altered metabolic parameters develop insulin resistance, which leads to the impairment of glucose metabolism in the brain and subsequently neuronal loss and neurological dysfunctions.^7,9^ More recently, other investigations have associated the development of AD with mitochondrial dysfunctions and glycolysis alterations in animals and humans.^10,11^ Still the molecular mechanisms behind obesity and brain dysfunctions remain poorly understood.

Since obesity is a complex and multifactorial pathology, it is proposed that multiple signalling pathways are contributing to its development. Protein-protein interactions and post-translational modifications (PTMs), such as protein phosphorylation, play crucial roles in signalling pathways. Nevertheless, little is known about the role of PTMs in the signalling pathway linking obesity to brain impairment. However, we previously showed that high-fat diet (HFD) as well as high-glycaemic diet (HGD) are changing the phosphoproteomic profile of many proteins involved in cytoskeletal re-arrangement and calcium homeostasis.^12^

Based on these lines of evidence, in this original work, we intent to investigate mouse brains proteomic changes, by focusing on different post-translational modifications, with the aim of shedding light on the signalling pathways linking diet-induced obesity and brain impairment.

Herein, a quantitative mass spectrometry-based proteomic approach was used to identify proteins and peptides that are significantly changing in mouse brains as a consequence of the different diets. The robust approach developed by Larsen and collaborators^11,12^ was used to identify and quantify phosphorylated peptides, N-linked sialylated glycopeptides and non-modified proteins. Data analysis revealed that peptides and proteins involved in metabolic and mitochondrial pathways were consistently changing in obese mice brains compared to mice fed with a standard diet (SD). PTMs site analysis also indicates that cellular respiration and cytoskeletal functionality are impaired in obese mice. Taken together, the data presented here, highlight the importance of PTMs, especially phosphorylation and glycosylation, in studying molecular pathways and might open future perspectives in the discovery of mechanisms underlying brain impairment and obesity.

## Material and Methods

### Animal model and in vivo measurement

The animal model and the in vivo measurements are reported in our previous study.^12^ Briefly, 3 groups, composed of 3 animals each, were fed respectively with high-fat diet (HFD) (5.2 kcal/g) (D12492, Charles River), hyperglycaemic diet (HGD) (4.0 kcal/g) (D12329, Charles River) and the SD (3.9 kcal/g) (12450B, Charles River) for 12 weeks. In vivo measurements were performed before animals were weighed and sacrificed by cervical dislocation. Animal brains were immediately explanted, weighed and stored at −80°C before further analysis.

### Protein and peptide preparation for MS

**Figure 1.**
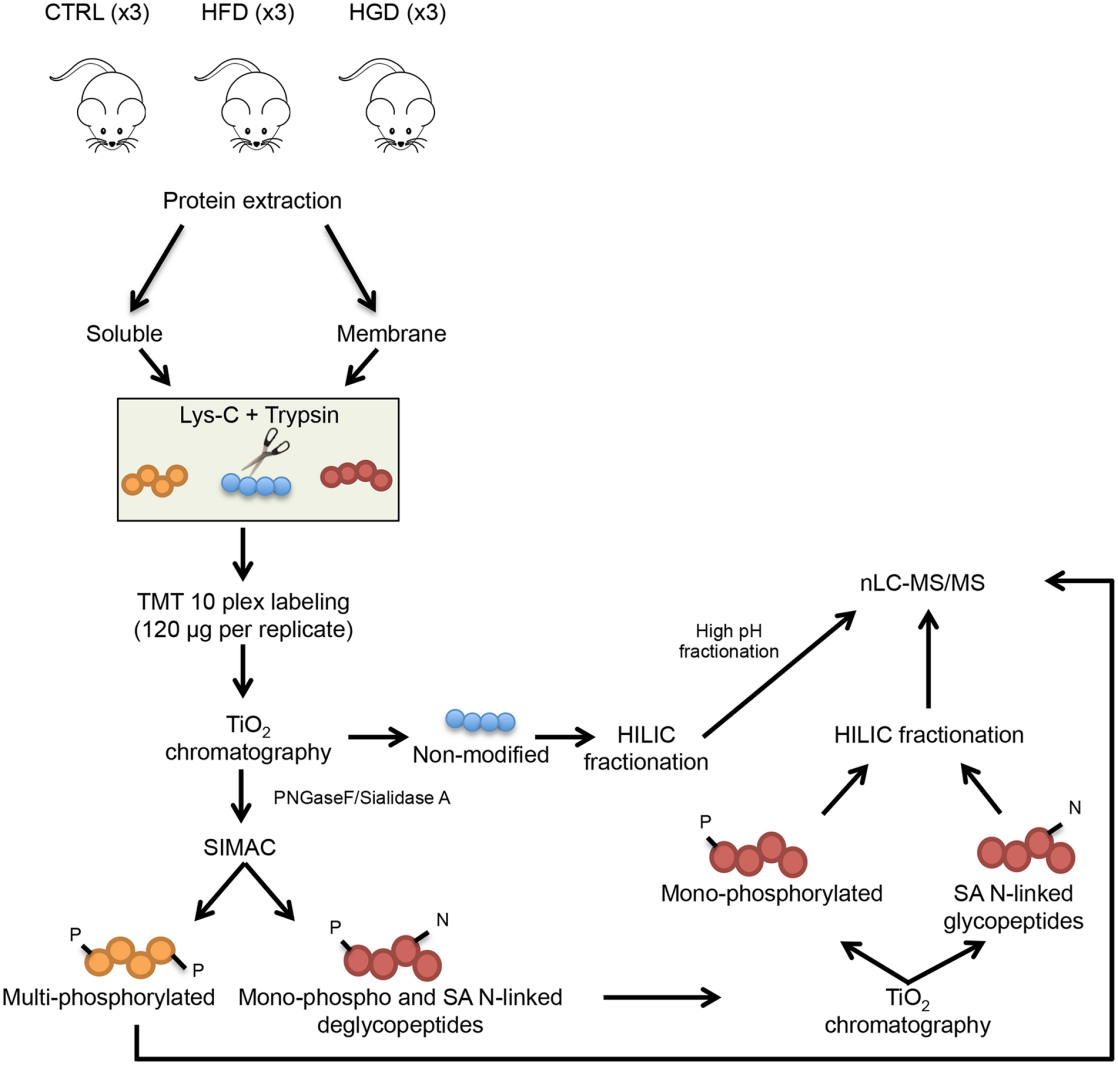
Workflow of quantitative mass spectrometry-based proteomic approach. After mice sacrifice, brains were collected from 3 mice fed with a standard diet (SD), 3 mice fed with high-fat diet (HFD) and 3 mice fed with high-glycaemic diet (HGD) and proteins were extracted. Soluble and membrane protein fractions were separated by ultracentrifugation followed by reduction, alkylation and digestion. Both fractions were TMT labelled and combined. Multiple enrichment steps using TiO_2_ and sequential elution from IMAC (SIMAC) beads resulted in 3 proteomic pools: non-modified peptides, phosphorylated peptides and formerly sialylated (SA) N-linked glycopeptides. Complexity of samples was reduced using HILIC fractionation for non-modified, mono-phosphorylated and SA N-linked peptides. Moreover, non-modified peptides were also fractionated by high pH and each fraction was run by nLC-MS/MS.

About 50 mg of brain powder was used for protein extraction. After ultracentrifugation, proteins were divided into membrane and soluble proteins for further analysis. Both protein fractions were TMT labelled and separately digested into peptides. Phosphopeptides and sialylated N-linked glycopeptides were enriched using multiple rounds of TiO_2._ The non-modified peptides and the enriched fractions were further fractionated (to increase peptides coverage) using high pH chromatography prior to LC-MS/MS analysis (see schematic workflow in Figure 1). A detailed material and method for protein extraction, TMT labelling, peptides enrichment and fractionation is described in Supplemental File S1.

### Reversed-phase nanoLC-ESI-MS/MS

The samples were resuspended in 0.1% formic acid (FA) and loaded onto a 2-column EASY-nano liquid chromatography (nLC) system (Thermo Scientific). The pre-column was a 3 cm long fused silica capillary (100 µm inner diameter) with a fritted end and in-house packed with ReproSil-Pur C18 AQ 5 µm whereas the analytical column was a 17 cm long fused silica capillary (75 µm inner diameter) and packed with ReproSil-Pur C18 AQ 3 µm reversed-phase material (both resins Dr. Maisch Ammerbuch-Entringen).

The peptides were eluted with an organic solvent gradient from 100% phase A (0.1% FA) to 34% phase B (95% ACN, 0.1% FA) at a constant flowrate of 250 nL/minute. Depending on the samples based on the HILIC, the gradient was from 1% to 28% solvent B in 50 or 80 minutes, 28% to 45% solvent B in 10 minutes, 45% to 100% solvent B in 5 and 8 minutes at 100% solvent B.

The nanoLC was online connected to a Q-Exactive HF Mass Spectrometer (Thermo Scientific) operated at positive ion mode with data-dependent acquisition. The Orbitrap acquired the full MS scan with an automatic gain control (AGC) target value of 3 × 10^6^ ions and a maximum fill time of 100 ms. Each MS scan was acquired at high-resolution (120 000 full width half maximum (FWHM)) at 200 m/z in the Orbitrap with a mass range of 450 to 1400 Da. The 15 most abundant peptide ions were selected from the MS for higher energy collision-induced dissociation (HCD) fragmentation (collision energy: 34 V). Fragmentation was performed at high resolution (60 000 FWHM) for a target of 1 × 10^5^ and a maximum injection time of 200 ms using an isolation window of 1.2 m/z and a dynamic exclusion for 30 seconds. All raw data were viewed in Thermo Xcalibur v3.0.

### Data analysis

Raw data were processes using Proteome Discoverer (v2.1, ThermoFisher) and searched against the Swissprot mouse database using an in-house Mascot server (v2.3, Matrix Science Ltd.) and the Sequest HT search engine. Database searches were performed with the following parameters: precursor mass tolerance of 10 ppm, fragment mass tolerance of 0.03 Da (HCD fragmentation), TMT 10-plex (Lys and N-terminal) as fixed modifications and a maximum of 2 missed cleavages for trypsin. Carbamidomethylation of alkylated cysteines along with phosphorylation of Ser/Thr/Tyr and deamidation of Asp for the phosphorylated and SA N-linked glycosylated groups, respectively, were set as variable modifications. All identified peptides were filtered against a decoy database, using Percolator with a false discovery rate (FDR) of 0.01 (FDR < 0.01). Only peptides with Mascot rank 1 and cut-off value of Mascot score >18 and a SEQUEST HT ∆Cn of 0.1 were considered for further analysis. Only proteins with more than 1 unique peptide were considered for further analysis in the non-modified group. For the modified peptides, formerly SA N-linked glycopeptides were filtered for the consensus motif NXS/T/C; X≠P and only proteins associated with membranes were considered. The relative abundances of modified peptides (phosphorylated and formerly glycosylated peptides) were normalised by the protein expression to distinguish differential regulated modifications from altered protein expression.^13^

### Statistical analysis

Statistical analysis (ANOVA) was performed for phosphorylated, formerly SA N-linked glycopeptides and non-modified peptides (*P*-value <.05) using Qlucore Omics Explorer 3.2 (Qlucore AB, Lund, Sweden). In addition, a fold change (FC) cutoff was further applied. FC was calculated as the ratio between HFD/SD and HGD/SD and values are reported as log2 transformed. Peptides and proteins in a range −0.5 < FC > 1.5 were considered significant. STRING pathway analysis^14^ was used to determine protein-protein interactions. A medium confidence interaction score (>0.4) was set and the following sources to retrieve interactions were used: text mining, experiments and databases. No more than 10 interactors (no query proteins) were allowed.

## Results and Discussion

The present study aimed at revealing molecular signalling mechanisms linking obesity and neuro-impairment by investigating the brains of mice fed with obesogenic diets compared to standard diet (SD). A total of 3 mice per group were investigated: control mice fed with a SD, mice fed with HFD and mice fed with HGD. To detect diet-mediated changes in the mouse brains, we performed large-scale quantitative phosphoproteomics, SA N-linked glycoproteomics and global proteomics by combining TMT-10plex quantitation, TiO_2_ enrichment of phosphopeptides and SA N-linked glycopeptides with high-accuracy and high-resolution nLC-MS/MS.

### The effect of diet-induced obesity on metabolic parameters

After 12 weeks of diet (HFD, HGD or SD) administration, metabolic parameters were measured. As reported in our previous study,^12^ mice fed with HFD or HGD showed a significant increase in basal glycaemia compared to SD fed mice. The HFD and HGD fed mice also showed an increase in body weight compared to SD fed mice, however, this did not reach to statistically significant levels. No differences were observed in food intake. These results are in accordance with other studies on mouse strain C57BL/6J showing the development of obesity and hyperglycaemia after HFD administration.^15,16^

### Quantification of PTMs and non-modified mouse brain proteome

Brains were collected and after protein extraction, soluble and membrane protein fractions were separated by ultracentrifugation. In solution digestion (using Lys-C and trypsin) was followed by TMT labelling and TiO_2_, SIMAC and HILIC chromatography were performed to separate and fractionate non-modified, phosphorylated and formerly SA N-linked glycopeptides prior to nLC-MS/MS analysis (Figure 1).

**Figure 2.**
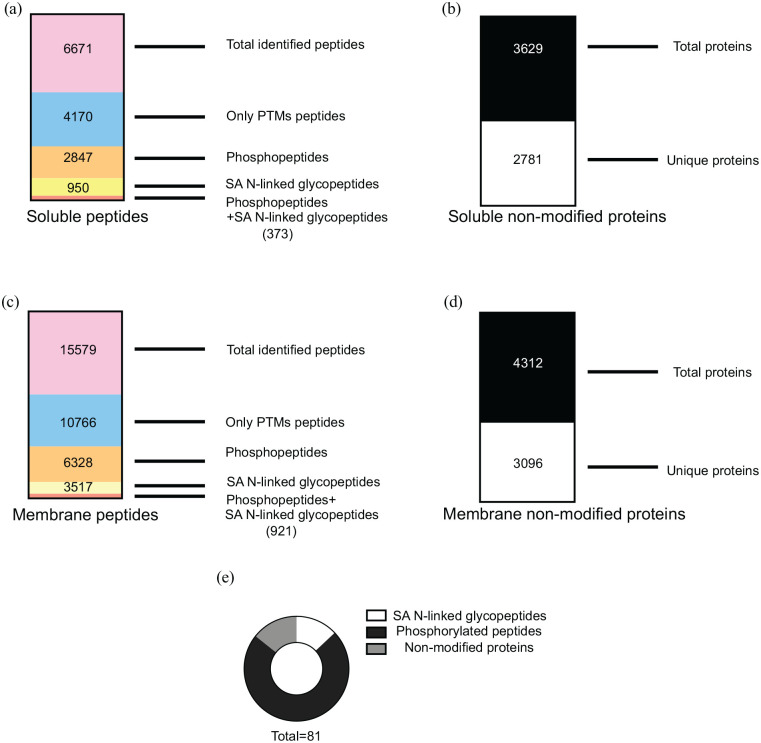
(a) MS/MS data analysis for the soluble fraction was performed using Proteome Discoverer 2.1. A total number of 6671 peptides were detected. Multiple filtering using Excel was applied and 4170 post-translational modified peptides (PTMs) were found. Among those 2847 phosphopeptides and 950 SA N-linked glycopeptides were detected while 373 peptides contained both modifications in the same sequence, (b) from Proteome Discoverer 2.1 a list of soluble non-modified proteins was exported. A total number of 3629 proteins were detected and 2781 proteins represented by 2 or more peptides were filtered out and used for further analysis, (c) MS/MS data analysis for the membrane fraction was performed using Proteome Discoverer 2.1. A total number of 15 579 peptides were detected. Multiple filtering using Excel was applied and 10 766 PTMs were found. Among those 6328 phosphopeptides and 3517 SA N-linked glycopeptides were detected while 921 peptides contained both modifications in the same sequence, (d) from Proteome Discoverer 2.1 a list of membrane non-modified proteins was exported. A total number of 4312 proteins were detected and 3096 proteins represented by 2 or more peptides were filtered out and used for further analysis, and (e) cake-graph for significant peptides and proteins in the dataset. Statistical analysis (univariate ANOVA test) was performed for each subset of peptides in the dataset and 81 peptides were significantly changing in the high-fat diet (HFD) or high-glycaemic diet (HGD) compared to standard diet (SD) (*P*-value < .05 and −0.5 < FC > 1.5). The graph is showing the proportions between SA N-linked glycopeptides (white), phosphorylated peptides (black) and non-modified proteins (grey).

Database search of the modified samples led to the identification of 6671 and 15 579 total peptides in the soluble and membrane fractions, respectively. Relative abundances of modified peptides (phosphorylated and formerly SA N-linked glycosylated peptides) were normalised to the protein expression to distinguish differentially regulated modifications. Given so, 2847 and 950 phosphopeptides and SA N-linked glycopeptides were identified in the soluble fraction, respectively; 6328 and 3517 phosphopeptides and SA N-linked glycopeptides were identified in the membrane fraction, respectively (Figure 2a and c). The non-modified fraction was analysed separately. A total of 2781 proteins represented by 2 or more unique peptides and 3096 represented by 2 or more unique peptides were identified in the soluble and membrane, respectively (Figure 2b and d). These data represent successful PTM enrichments of soluble and membrane and their non-modified counterparts fractions on mice brain proteins. Full list of peptides and proteins identified can be found in Supplemental File S2.

### Diet, PTMs and non-modified proteome patterns

ANOVA test was performed to identify significant changes in peptides and proteins between HFD, HGD and SD. A *P*-value <.05 and a fold change (FC) lower than −0.5 and higher than 1.5 was used to filter the data. Following these parameters 69 peptides (57 phosphorylated peptides and 12 glycosylated peptides) were identified, while 12 non-modified proteins were found to be significantly altered (Figure 2e). The full list of the significant peptides and proteins is reported in Supplemental File S3.

In this dataset the phosphorylation changes mainly occurred in cytoskeletal proteins. This result is not surprising since cytoskeletal proteins are highly abundant and highly phosphorylated also in basal conditions.^17-19^ On the contrary, fewer proteins were found to be glycosylated. The glycosylated proteome is less abundant and technically more difficult to detect.^20^ All the N-linked glycopeptides have less sialic acid in both mice fed with HFD and HGD.

**Figure 3.**
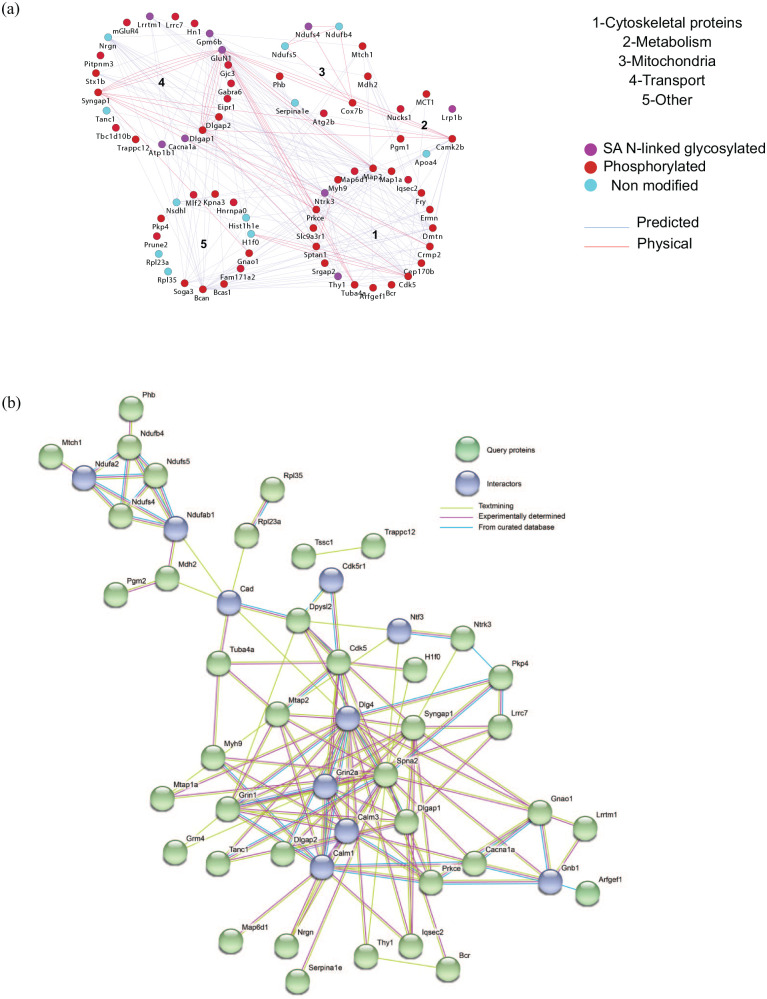
(a) Cytoscape GO protein cluster map. Each dot in the map represent a protein and each colour represent peptide modifications: glycosylation (purple), phosphorylation (red) and non-modified (turquoise). Cluster 1 contains 19 proteins involved in cytoskeletal functions; cluster 2 contains 5 proteins involved in metabolism; in cluster 3 are grouped 8 proteins involved in mitochondrial functionality; cluster 4 contains 14 proteins involved in transport, while cluster 5 contains 10 proteins involved in different biological processes which could not be clustered in a common category and (b) STRING interaction map showing query nodes (green) and interaction nodes (blue). Interaction score was set as medium confidence (>0.4) and the interactions displayed are based on the following sources: text mining (yellow line), experimentally determined (pink line), curated database (turquoise line).

Based on the statistical analysis, we decided to further look into the proteins that are significantly changing in the dataset. Thus, we performed a Gene Ontology (GO) analysis for all the significantly changed peptides and proteins. Figure 3a shows protein clusters, based on GO using Cytoscape (version 3.5.1)^21^, node colours represent peptide modifications while edges represent the interaction between nodes. Cluster 1 contains 20 proteins involved in cytoskeletal functions; cluster 2 contains 6 proteins involved in metabolism; in cluster 3 are grouped 9 proteins involved in mitochondrial functionality; cluster 4 contains 20 proteins involved in transport, while cluster 5 groups 15 proteins involved in different biological processes, which do not cluster in a common category. Furthermore, we visualised the interaction network of the significant phosphorylated, N-linked sialylated peptides and non-modified protein using STRING.^14^ In STRING we could include ‘interactor’ nodes (proteins), closely related to query nodes and visualise interactions coming from text mining, experimental determinations and curated databases. Excluding the disconnected nodes, most of these interactions are connecting proteins involved in transport with cytoskeletal proteins (Figure 3b).

### Analysis of the interaction pathways

Cytoskeleton is the major component of cells in vertebrates. It regulates the motility of proteins in the cells and abnormal changes of cytoskeleton have been linked to pathological conditions.^22^ Since we already analysed the role of cytoskeletal proteins in obese mice brains in our previous study, we did not further focus on the cytoskeleton for the interaction pathway analysis here. In fact, we previously showed significant changes in the phosphorylation status of cytoskeletal proteins such as glycogen synthase kinase 3 beta (GSK3β), adducins and myristoylated alanine-rich C-kinase substrate (MARCKS).^12^ Analysis of phosphosites revealed the inability of these proteins to maintain cytoskeletal integrity and their capacity of starting microtubules (MTs) disassembly. In the present work, only some of the proteins involved in the cytoskeletal network will be further discussed. Considering the novelty of this work, we found of better interest to analyse and discuss proteins found in the dataset that belong to the 2 clusters called ‘metabolism’ and ‘mitochondria’.

Changes in proteins involved in the metabolic pathway are foreseen since our previous in vivo results showed changes in metabolic parameters such as increase of fasting glycaemia and increase in body weight for HFD and HGD fed mice compared to SD fed mice.^12^ Proteins such as monocarboxylate transporter 1 (MCT1) and phosphoglucomutase-1 (PGM1) are important for the maintenance of glucose homeostasis. For instance, mice fed with HGD exhibit a reduced phosphorylation of PGM1 (S117). This site is the active site for magnesium binding and its phosphorylation by glucose-1,6-biphoshate enhances PGM1 activity.^23^ This data suggests a reduced glucose breakdown leading to impaired glucose metabolism in HGD compared to SD. Furthermore, an alteration of MCT1 expression (a significant reduced phosphorylation was found in HFD fed mice) could lead to metabolic disorders since MCT1, in physiological conditions, facilitates the passive transport of monocarboxylates such as lactate, pyruvate and ketone bodies together with protons across cell membranes.^24^

Interestingly, mitochondria seem to be affected by the diet. In our dataset, 9 proteins involved in mitochondrial activity are significantly changing. In particular dehydrogenase [ubiquinone] iron-sulphur protein 4 and 5 (NDUFS4 and NDUFS5), and cytochrome c oxidase subunit 7B, mitochondrial (COX7) (components respectively of complex I and complex IV of the respiratory chain) show a lower expression in obese compared to lean mice. It is well established that changes in the mitochondrial respiration chain cause catastrophic effects on the functionality of the cells and can lead to metabolic disorders.^11,25^

Cluster 4 (shown in Figure 3a) includes proteins involved in all sorts of transport such as ion channels and molecules associated to ion channels like: sodium/potassium-transporting ATPase subunit alpha-1 (ATP1A1), voltage-dependent P/Q-type calcium channel subunit alpha-1A (CACNA1A), disks large-associated protein 2 (DLGAP2); as well as neurotransmitters receptors like: neuronal membrane glycoprotein M6-b (GPM6B), glutamate receptor ionotropic, NMDA 1 (GLUN1) and metabotropic glutamate receptor 4 (mGLUR4). Most of the proteins involved in transport show a reduced sialylation in mice fed with HFD and/or HGD.

Other proteins like syntaxin1a (STX1A), GLUN1, neurogranin (NRGN) as well as the ion channels like CACNA1A are involved in calcium (Ca^2+^) transport. Ca^2+^ plays a central role in the regulation of many different pathways and an impairment on Ca^2+^ homeostasis can lead to cellular dysfunctions.^26,27^ In the STRING pathway enrichment, proteins such as calmodulin-1, calmodulin-3 and Grin2a have a pivotal role in the interaction network. These ‘interactors’ are connecting ion channels and cytoskeletal proteins, suggesting that diet is affecting the transport of Ca^2+^ and glutamate in neurons. Some of the proteins discussed above can be found in Table 1 and a complete list of the proteins is reported in Supplemental File S3.

**Table 1.** Some of the significant proteins involved in pathways known to be implicated in obesity and/or neuronal loss, such as glucose homeostasis, mitochondrial respiration, ion channels and neurotransmission.

Protein accession number	Protein name	*P*-value	Fold change (HFD/SD)	Fold change (HGD/SD)	Function/Biological process
P53986	Monocarboxylate transporter 1	0.03	−0.72	−0.06	Glucose homeostasis
Q9D0F9	Phosphoglucomutase-1	0.01	0.08	−0.64	Glucose homeostasis
Q9CXZ1	NADH dehydrogenase [ubiquinone] iron-sulphur protein 4	0.01	−0.39	−0.71	Mitochondrial respiration
Q99LY9	NADH dehydrogenase [ubiquinone] iron-sulphur protein 5	0.01	−0.4	−0.47	Mitochondrial respiration
P56393	Cytochrome c oxidase subunit 7B	0.02	−0.66	−0.91	Mitochondrial respiration
P14094	Sodium/potassium-transporting ATPase subunit beta-1	0.03	−0.08	0.06	Ion channels
P97445	Voltage-dependent P/Q-type calcium channel subunit alpha-1A	0.02	−0.57	−0.25	Ion channels
Q8BJ42	Disks large-associated protein 2	0.002	0.55	0.17	Ion channels
P16305-1	Gamma-aminobutyric acid receptor subunit alpha-6	0.03	−0.84	−0,56	Neurotransmitters receptor
P35438	Glutamate receptor ionotropic, NMDA 1	0.03	−1.34	−0.3	Neurotransmitters receptor
P35803	Neuronal membrane glycoprotein M6-b	0.02	−0.94	−0.78	Neurotransmitters receptor
Q68EF4	Metabotropic glutamate receptor	0.03	−0.63	−0.54	Receptor

The table is reporting *P*-values, fold changes (as ratios HFD/SD and HGD/SD) as well as the function/biological process of the listed proteins.

### Obesity and neurodisorders markers

**Figure 4.**
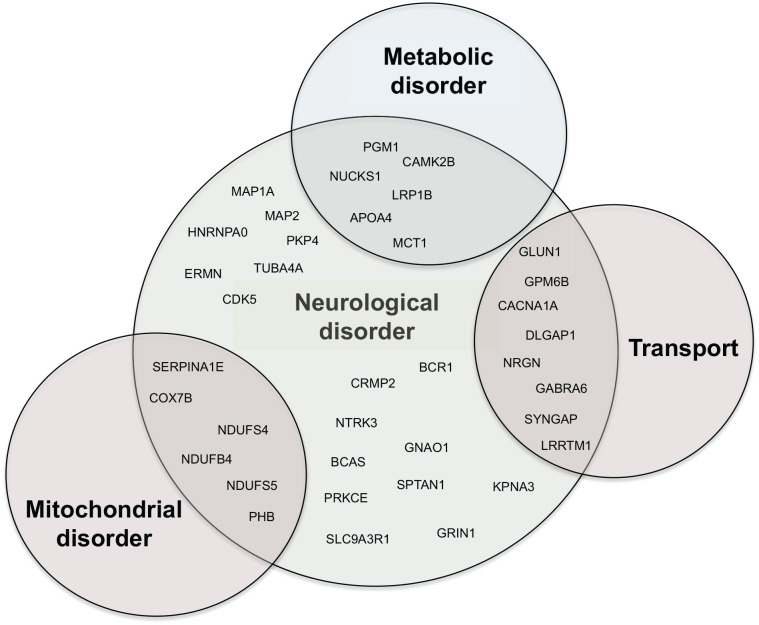
Venn diagram showing proteins involved in neurological disorders. GO analysis and literature mining revealed that 37 proteins in the dataset have been previously reported as implicated in neurological disorders. Among those, 6 are also involved in mitochondrial disorders, 5 are involved in metabolic disorders and 7 are part of the transport machinery.

Among the 77 significant changing peptides and proteins grouped in 5 different clusters, we questioned how many of those could be involved in brain impairment. Indeed, it is established that a chronic metabolic disorder with development of obesity and hyperinsulinaemia can lead to brain impairment.^9,28^ Using STRING and by literature mining, we identified, in our dataset, 37 proteins that are associated to different kinds of brain impairment and brain disorders. These proteins involved in metabolic and mitochondrial processes as well as cytoskeletal mechanisms and transport also play a role in brain impairment (Figure 4). Some of these proteins (expressed as intensities in the graphs) are depicted in the histograms in Supplemental Figure S1.

Firstly, the de-phosphorylation of PGM1 protein (S117) and the reduced expression of MCT1 cause a reduction of glycolysis, a central mechanism for energy production in the brain. Reduction of MCT1 impairs learning and memory as well as nerve impairment in murine models.^29^ Taken together this data support that alteration of metabolism in the brain could lead to brain impairment and brain disorders.^30^

Secondly, mitochondrial activity is extremely important for maintaining cell homeostasis especially in the brain. In general a reduction of mitochondrial complexes expression causes a reduced mitochondrial energy metabolism. We have found a reduced phosphorylation of COX7b, NDUFS4, NDUFS5 and MTCH1. All these proteins phosphorylation changes were never reported before, but theirs reduced expression at the protein level was observed in post-mortem brain of patients with AD.^31^ We also observed an increased phosphorylation of malate dehydrogenase, mitochondrial (MDH2) protein, which leads to inhibition of its enzymatic activity^32^ and subsequent inhibition of Krebs cycle. Indeed, the reduction of MDH2 activity causes an impairment of Krebs cycle and subsequent reduction of ATP production,^33^ necessary for mitochondrial respiration, thus, leading, most likely to catastrophic effects in neurons.

The impairment of mitochondrial activity also causes the production of reactive oxygen species (ROS), one of the main causes of brain damage. ROS contribute to the development of various brain disorders such as AD and Parkinson’s disease.^34^ Moreover, in our dataset a reduced sialylation for all the membrane proteins was observed in obese mice. It has previously been reported that reduced global glycosylation of proteins in the brain is associated with low brain glucose metabolism and impaired glucose metabolism is one of the main factors for the development of AD.^35^

Thirdly, increased expression of NRGN in both HFD and HGD fed mice might be another factor of brain function impairment. Indeed, changes in this protein expression have been also linked to AD development.^36^ Additionally, an increased level of Apolipoprotein A-IV (APOA4) was observed in HFD fed mice. This protein plays a consistent role in feeding behaviour, in the sense of satiety and in brain metabolism and has been found overexpressed in AD patients.^37,38^

Fourthly, the correlation between cytoskeletal changes and brain impairment has been well established for many years now.^39,40^ In our dataset a large number of the cytoskeletal proteins are phosphorylated (Figure 2e) and some of the phosphosite changes are well characterised. For example, we detected an increased phosphorylation of dihydropyrimidinase-related protein 2 (CRMP2) in multiple sites (S507/T509/T512/T514 and S540) in both HFD and HGD fed mice. Multiple phosphorylation sites of CRMP2 have been related to neuronal impairment and schizophrenia^41^ and hyperphosphorylation of this protein has been proposed as an early AD biomarker.^42,43^ Another example is given by the increased phosphorylation of the microtubule-associated protein 2 (MAP2) (S1783 and S1791) in mice fed with HFD. MAP2 is one of the most studied microtubule-associated proteins and its phosphorylation causes the detachment from microtubules and subsequent MTs destabilisation with strong consequences in synaptic transmission,^44,45^ which could further lead to neurological disorders.^22^

## Conclusions

The present study aims to explore proteomic changes in the brain of obese mice to investigate pathways that might explain and reveal the connection between diet-induced obesity and neuronal impairment. For this purpose, a quantitative mass spectrometry-based approach for the identification and quantification of post-translational modified proteins (phosphorylated and N-linked glycosylated) was employed. We have identified several peptides and proteins that changed in their expression levels in brains of mice fed with 2 different kind of obesogenic diets (HFD and HGD) compared to standard diet (SD). Albeit protein cellular functions are diverse, 3 features clearly emerged: (1) The normal function of mitochondrion is severely affected, (2) there is an alteration of expression levels of cytoskeletal protein, and (3) metabolic pathway proteins are altered. Several proteins detected here have been already reported to be associated with neurological disorders such as psychiatric disease, Alzheimer’s disease and mood disorders. These data suggest that neuronal loss is a complex process that involves proteins implicated in many pathways and that obesity is influencing many of the aspects that contribute to neurological disorders development. Taken together these data highlight new protein-protein interactions and novel protein modifications that are implicated in obesity and metabolic disorder as well as brain impairment. Even though, due to a limited amount of material, we could not analyse the brains with other techniques, we believe that our data open new perspectives for the study of molecular mechanisms relating obesity to brain disorders and further studies would benefit from the findings reported here.

## Supplemental Material

sj-docx-1-nmi-10.1177_11786388211012405 – Supplemental material for Obesogenic Diets Cause Alterations on Proteins and Theirs Post-Translational Modifications in Mouse Brains Click here for additional data file. Supplemental material, sj-docx-1-nmi-10.1177_11786388211012405 for Obesogenic Diets Cause Alterations on Proteins and Theirs Post-Translational Modifications in Mouse Brains by Valentina Siino, Pia Jensen, Peter James, Sonya Vasto, Antonella Amato, Flavia è, Giulia Accardi and Martin Røssel Larsen in Nutrition and Metabolic Insights

sj-xlsx-2-nmi-10.1177_11786388211012405 – Supplemental material for Obesogenic Diets Cause Alterations on Proteins and Theirs Post-Translational Modifications in Mouse Brains Click here for additional data file. Supplemental material, sj-xlsx-2-nmi-10.1177_11786388211012405 for Obesogenic Diets Cause Alterations on Proteins and Theirs Post-Translational Modifications in Mouse Brains by Valentina Siino, Pia Jensen, Peter James, Sonya Vasto, Antonella Amato, Flavia è, Giulia Accardi and Martin Røssel Larsen in Nutrition and Metabolic Insights

sj-xlsx-3-nmi-10.1177_11786388211012405 – Supplemental material for Obesogenic Diets Cause Alterations on Proteins and Theirs Post-Translational Modifications in Mouse Brains Click here for additional data file. Supplemental material, sj-xlsx-3-nmi-10.1177_11786388211012405 for Obesogenic Diets Cause Alterations on Proteins and Theirs Post-Translational Modifications in Mouse Brains by Valentina Siino, Pia Jensen, Peter James, Sonya Vasto, Antonella Amato, Flavia è, Giulia Accardi and Martin Røssel Larsen in Nutrition and Metabolic Insights
