# Supplementary material for: Obesogenic Diets Cause Alterations on Proteins and Theirs Post-Translational Modifications in Mouse Brains
Source: Nutr Metab Insights. 2021 May 3;14:11786388211012405. doi: 10.1177/11786388211012405 (PMC8114309; doi:10.1177/11786388211012405)
Supplement: sj-docx-1-nmi-10.1177_11786388211012405 – Supplemental material for Obesogenic Diets Cause Alterations on Proteins and Theirs Post-Translational Modifications in Mouse Brains [file sj-docx-1-nmi-10.1177_11786388211012405.docx]

Supplementary file S1

Mass spectrometry extended material and method

*Protein extraction and TMT labeling*

Mouse brain tissue was pulverized in dry ice and liquid nitrogen, using a Micro-dismembrator II (Braun Biotech International, Melsungen, Germany). A total of 50 mg of brain powder was dissolved in 1 mL of ice-cold 0.1 M Na2CO3, pH 11 supplemented with protease, phosphatase inhibitors (Sigma) and 10 nM sodium pervanadate. The solution was sonicated 2x20 seconds (Merck Millipore) and then incubated at 4°C for 1 hour. The supernatant was transferred to a 4 ml ultracentrifuge vial (Beckman Culter XPN-80) and ultracentrifugation (100,000xg) was performed for 90 minutes. Supernatant containing the soluble protein fraction was collected and the pellet containing the membrane protein fraction was transferred to a low binding tube. Soluble proteins were concentrated using 10kDa filters (AmiconULTRA) by 15 minutes centrifugation (14,000xg) at 4°C. Soluble proteins were re-dissolved and reduced using 6M Urea/2M Thiourea (Sigma) and 20 mM dithiothreitol (DTT) for 40 minutes, followed by alkylation with 50 mM Iodacetamide (IAA) for 40 minutes in the dark. Proteins were predigested with 2 µL Lys-C (Wako Pure Chemical Industries, Japan) for 3 hours at room temperature (RT) and the sample was diluted with 50 mM Triethyl Ammonium Bromide (TEAB) buffer (pH 7.5) (total volume added 200 µl).

Protein concentration measurement using Qubit (Thermo Scientific) was performed prior to protein digestion with trypsin (Promega) (2% w/w) overnight (ON) at 37°C. Formic acid (2% v/v) was subsequently added and the samples were centrifuged for 10 min at 14,000xg, to precipitate lipids from the solution.

Membrane proteins were dissolved in 6M Urea/2M Thiourea, reduced, alkylated and digested as described above. Thereafter, a total of 120 µg of the samples were labeled with Tandem Mass Tag (TMT) 10plex Isobaric label Reagents (Thermo Scientific) following the manufacturer's instructions. The labeled peptides were mixed 1:1:1:1:1:1:1:1:1 and dried by vacuum centrifugation.

*Enrichment of phosphopeptides and sialylated (SA) N-linked glycopeptides*

Enrichment of phosphopeptides and sialylated (SA) N-linked glycopeptides was performed using a modified TiSH procedure (13, 14) in which non-modified peptides are separated from the modified peptides using TiO2, and subsequently the multi- and monophosphorylated peptides are separated from formerly SA N-linked glycopeptides after a deglycosylation step using sequential elution from IMAC beads (SIMAC) procedure. Thereafter peptides are fractionated by hydrophilic interaction liquid chromatography (HILIC) (see schematic workflow in Figure 1).

Peptides were dissolved in 80% acetonitrile (ACN)/5% trifluoroacetic acid (TFA) with 1 M glycolic acid (Sigma) and incubated with 0.6 mg TiO2 beads (Titansphere 10 m, GL Sciences) per 100 µg peptide for 15 min at RT with vigorous shaking. The beads were briefly centrifuged and the supernatant transferred to a new tube with 0.3 mg TiO2 beads per 100 µg peptide. After 15 min incubation at RT with vigorous shaking and a brief centrifugation the supernatant was collected. Subsequently, the beads were washed with 80% ACN/1% TFA and 10% ACN/0.1% TFA. The supernatant with the unbound TiO2 fraction and the washing fractions, both containing the non-modified peptides, were combined. The phosphorylated peptides were eluted from the beads by incubation with 1.5% ammonium hydroxide solution (Sigma), pH 11.3, at RT with vigorous shaking for 15 min. Samples were centrifuged and the supernatant passed through a C8 filter from a 3M EmporeTM disk (Sigma). Any remaining peptides were eluted from the disk with 30% ACN and all peptide samples were lyophilized.

To separate the SA N-linked glycopeptides that also bind to the TiO2 beads (15), the dried sample was deglycosylated with N-glycosidase F (Biolabs) and Sialidase A (Prozyme) at 37°C ON. The sample was dried, resuspended in SIMAC loading buffer (50% ACN/0.1% TFA) and incubated with PhosSelect IMAC beads (Sigma), pre-equilibrated in SIMAC loading buffer for 30 min at RT with gentle shaking. The beads were packed in a constricted p200 GeLoader tip by applying gentle air pressure with a 1 ml syringe and the flow through collected and combined with the washing collected from the beads (SIMAC loading buffer) before mono-phosphorylated and deglycosylated peptides were eluted with 20% ACN/1% TFA and combined with the washing fraction and dried. The multi-phosphorylated peptides were eluted with 1.5% ammonium hydroxide solution, pH 11.3, and dried. Mono-phosphorylated and deglycosylated peptides were separated by adjusting the sample to 70% ACN/2% TFA and repeating the TiO2 bead enrichment as described above.

*Desalting procedure*

All the eluates were dried and desalted on self-made P200-tip-based columns using only R3 material for the mono- and multi-phosphorylated peptides and R2/R3 material for deglycosylated-peptides and non-modified proteins. The samples were re-dissolved and acidified with 0.1% TFA (Sigma). A small plug of C18 material from a 3M EmporeTM disk (Sigma) was inserted in the constricted end of a P200 pipette tip and 1.5 cm of the tip was packed with reversed-phase resin material consisting of a 1:1 mix of Poros 50 R2 and Oligo R3 (both Applied Biosystems) resins or only R3 resin dissolved in 100% ACN (Sigma) by applying air pressure with a 1 ml syringe. The acidified samples were loaded onto the micro-column and washed with 0.1% TFA. The peptides were eluted using 60% ACN/0.1% TFA before the samples were dried by vacuum centrifugation.

*Hydrophilic interaction liquid chromatography (HILIC) and high pH fractionation*

To reduce sample complexity mono-phosphorylated, deglycosylated and non-modified peptides were fractionated using HILIC as previously described (Engholm-Keller et al. 2012). The non-modified sample was first diluted in 0.1% TFA and approximately 50 µg peptide was fractioned. All mono-phosphorylated and deglycosylated samples were fractionated. The samples were dissolved in 90% ACN, 0.1% TFA (solvent B) and loaded onto an in-house packed TSKgel Amide-80 (Tosoh Bioscience) micro-capillary column (450 μOD x 320 μID x 17 cm) using an Agilent 1200 Series HPLC (Agilent). Peptides were separated using a gradient from 100-60% solvent B (A = 0.1% TFA) running for 30 min at a flow-rate of 6 µl/min. Fractions were collected every 1 min and combined into 12-15 final fractions based on the UV chromatogram and subsequently dried by vacuum centrifugation.

To increase the coverage of the non-modified peptides, off-line high pH fractionation on a microcolumn was also performed using approximately 50 µg peptide. Briefly, the sample was dissolved in 1% ammonium hydroxide (NH3, Sigma), pH 11, and loaded on a R2/R3 column equilibrated with 0.1% NH3. The peptides were eluted in a stepwise fashion using a gradient of 5%-60% ACN/0.1% NH3. All fractions were dried by vacuum centrifugation and stored at -20°C prior to LC-MS/MS analysis.
